# The potential of incorporation of binary salts and ionic liquid in P(VP-co-VAc) gel polymer electrolyte in electrochemical and photovoltaic performances

**DOI:** 10.1038/srep27630

**Published:** 2016-06-08

**Authors:** Ng Hon Ming, S. Ramesh, K. Ramesh

**Affiliations:** 1 Centre for Ionics University of Malaya, Department of Physics, Faculty of Science, University of Malaya, Kuala Lumpur, Malaysia

## Abstract

In this study, dye-sensitized solar cells (DSSCs) has been assembled with poly(1-vinylpyrrolidone-co-vinyl acetate) (P(VP-co-VAc)) gel polymer electrolytes (GPEs) which have been incorporated with binary salt and an ionic liquid. The potential of this combination was studied and reported. The binary salt system GPEs was having ionic conductivity and power conversion efficiency (PCE) that could reach up to 1.90 × 10^−3^ S cm^−1^ and 5.53%, respectively. Interestingly, upon the addition of the ionic liquid, MPII into the binary salt system the ionic conductivity and PCE had risen steadily up to 4.09 × 10^−3^ S cm^−1^ and 5.94%, respectively. In order to know more about this phenomenon, the electrochemical impedance studies (EIS) of the GPE samples have been done and reported. Fourier transform infrared studies (FTIR) and thermogravimetric analysis (TGA) have also been studied to understand more on the structural and thermal properties of the GPEs. The Nyquist plot and Bodes plot studies have been done in order to understand the electrochemical properties of the GPE based DSSCs and Tafel polarization studies were done to determine the electrocatalytic activity of the GPE samples.

In today’s world, energy requirement has become a hot topic in almost every nation around the world. This has boosted up the attention from the energy researcher communities to develop new types of materials and technologies for the energy production, storage, and conversion. Renewable energy resources are gathering a great number of interests as mankind would need to rely on the renewable energy in the upcoming decades as the fossil fuels that were being used today were getting depleted throughout the year^1^. Solar energy is one of the uprising renewable energy technology and could be a reliable choice to face growing energy demand from the population of our earth. This is because of the abundance of the sun energy (1004 Wm^−2^ at ground level with the sun directly overhead) that could be easily obtainable throughout the world^2^. With that reason alone, it has garnered a lot of researches to be done on the solar energy in the past few years.

Among the studies that have been reported, dye-sensitized solar cell (DSSC) is showing up as a promising solar harvesting technology that has bright future. This technology was invented by B. O’regan and M. Gratzel around two decades ago^3^. Being consisted merely just conductive glasses with a different layer of materials for anode and cathode, an organic or inorganic molecular dye, and an electrolyte which consists of redox couple^4^; these cells might be able to top the leading silicon-based solar cells in the upcoming years with a number of its own unique of promising properties. Moreover, with the ability that able to convert sunlight even under the low sunlight condition, these DSSCs could overcome one of the huge limiting factors of the silicon-based solar cells which is the capability of working only under perfect irradiation condition. This technology is really suitable for those countries which are having climates that are not suitable for the silicon-based technology solar cells^5^.

As stated previously, the DSSC composed of different types of components and materials. This has allowed groups of researchers to allocate themselves to study the DSSCs in a lot of different directions. Researchers who come from the different background could engage on different components to improve the photovoltaic performances of the DSSCs. Studies such as synthesizing new types of inorganic dyes^6^,^7^, incorporating new semiconductor layers^8^,^9^, replacing materials for counter electrodes^10^,^11^ and introduction of new redox couples have been done since the first report on DSSCs^12^,^13^. The research community finds the development of electrolyte is the hardest. The highest performing electrolytes for DSSCs up to date are the liquid electrolyte. An impressive photovoltaic conversion efficiency (PCE) of 12% has been achieved with liquid electrolytes based DSSCs but problems such as long term storage are hindering the development of these of DSSCs^14^. Due to this problem, researchers have started to work on different type of electrolytes and found that gel type of electrolytes have the potential to replace the conventional liquid type of electrolytes. There are a huge amount of advantages of using gel electrolytes over the liquid electrolytes in the application of DSSCs. One of it would be the improvement of the shelf time storage of the gel polymer electrolytes based DSSCs over the liquid electrolytes based DSSCs^15^,^16^,^17^. Wang *et al*. has reported that their P(VA-co-MMA)-based GPE were able to maintain the performances of their devices at 96% after 1000 hours^18^. Another group also reported that their Felmion-based gel electrolyte was able to sustain 90% of the efficiency of their DSSCs up to 4392 hours of storage time^19^. This is one of the main reasons for us to use gel polymer electrolytes as one of the main component for our DSSCs. Unfortunately, the PCE rate of the gel electrolytes were not even comparable due to various reasons such as the formation of the gel network that hindered the movement of the mobile ions in the system^20^. With that, a huge list of additives and materials has been proposed over the years to produce the best performing gel electrolytes but to the fact that there is still no optimum solution has been identified yet.

In this respect, we herein propose the incorporation of 1-methyl-3-propylimidazolium iodide (MPII) into the poly(1-vinylpyrrolidone-co-vinyl acetate) (P(VP-co-VAc)) based co-polymer electrolyte containing binary salts in order to produce a highly efficient gel polymer electrolyte (GPE) for the application of DSSCs. Co-polymers have both amorphous and crystalline phases which could provide plasticity and mechanical strength, respectively for our gel polymer electrolytes^21^,^22^ and binary salts system was found to have higher potential compared to the single salts system as reported by Dissanayake *et al*.^23^. Meanwhile, ionic liquids was widely known to be able to improve the electrical performances of the polymer electrolytes^24^,^25^. Even though there are a lot of studies done on the usage of copolymer, binary salt system and incorporation of ionic liquid to improve their performance, the combination of these three additives is yet to be studied extensively. With that in mind, P(VP-co-VAc) gel polymer electrolytes (GPE) with the incorporation of potassium iodide (KI), tetrapropylammonium iodide (TPAI) and 1-methyl-3-propylimidazolium iodide (MPII) were prepared, studied and reported.

## Results and Discussion

The overall solar to energy conversion efficiency is hugely dependent on the mobility of the redox couple and consequently, on the ionic conductivity of the polymer electrolyte for gel electrolyte based dye-sensitized solar cell devices^26^. With this reasons in mind, binary salts and MPII ionic liquid were added into the P(VP-co-VAc) based GPEs and the effects of these addition were studied with EIS technique.

**Figure 1:**
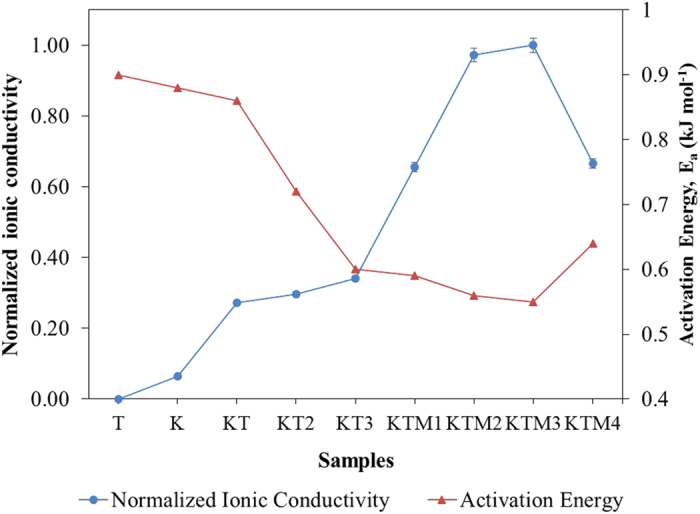
Variation of normalized ionic conductivity and activation energy for different GPE samples at room temperature.

Figure 1 shows the ionic conductivity at room temperature of the gel polymer electrolytes with the single salt system, binary salt system and system with the incorporation of MPII. The designation of the GPE samples was shown in Table 1. From the impedance measurement, the gel polymer electrolytes with single salt only exhibit ionic conductivities of 0.86 × 10^−3^ S cm^−1^ and 1.23 × 10^−3 ^S cm^−1^ for TPAI^27^ and KI salt system, respectively. However, as observed in Fig. 1, the GPEs with binary salt system shows higher ionic conductivity of 1.90 × 10^−3^ S cm^−1^ compared to the single salt systems. This is most likely due to the reason that the two cations were functioning in a different way inside polymer matrix and balancing out each other’s advantages appropriately. The larger TPA^+^ cations assisted in the enlargement of the polymer matrix to allow smaller K^+^ which already moving swiftly inside the polymer matrix to be able to move much faster and easier^28^. The ionic conductivity can then be seen further improved with the addition of MPII ionic liquid into the binary salts system to a value of 4.09 × 10^−3^ S cm^−1^. The addition of MPII helped in the enhancement of the dissociation process of the KI and TPAI salts. Furthermore, ionic liquid such as MPII was well known to have plasticizing effect which could soften the backbone of the host polymer matrix and increase the flow of the mobile charge movement which could lead to an increase in ionic conductivity. The decrease of the ionic conductivity after KTM3 was due to the agglomeration of the excess mobile charge ions from the MPII ionic liquids which could leads to formation of neutral pairs. These neutral pairs restricting the mobility of the mobile charge ions and decreases the ionic conductivity of the GPE sample^29^.

**Figure 2:**
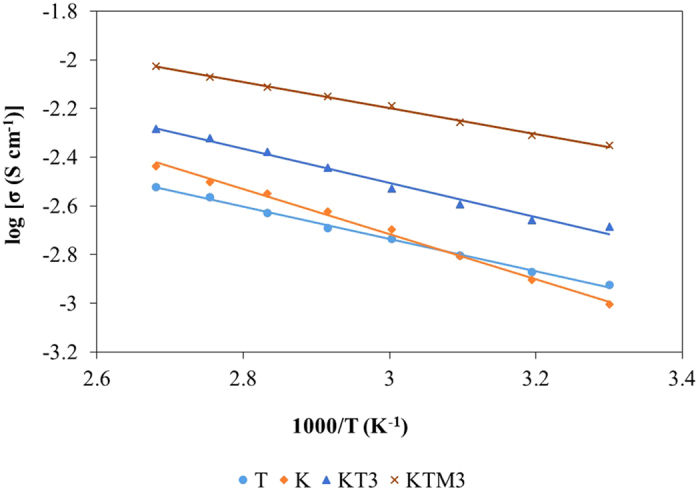
Arrhenius plots for the conductivity of the different GPE samples at room temperature.

The dependence of the ionic conductivity on the temperature, ranging from 333 K to 403 K of sample K, sample T, sample KT3, and sample KTM3 was shown in Fig. 2. From the figure, it can be seen that the conductivity increased with the increase of temperature and the log(σ) versus 1/T plots is close to unity for every GPE samples shown. This σ-T behavior for the gel electrolyte based samples can be described by the Arrhenius equation shown as below^30^:


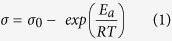


where *σ* is the conductivity, *σ*_*0*_ is the pre-exponential factor, *E*_*a*_ is the activation energy, *R* is the molar gas constant and *T* is the absolute temperature. Figure 1 shows the activation energy calculated from the slope of the data from the GPE samples from Fig. 2. As observed in the figure, sample KTM3 has the lowest *E*_*a*_ compared to other samples. This indicates that there were faster I^−^/I_3_^−^ transportation rate in the polymer matrix for sample KTM3 over the other samples^30^. Normally, when an ionic transport process involves intermolecular ion hopping, the conductivity is determined by the thermal hopping frequency, which in turn is proportional to the *exp(-E*_*a*_*/RT)*, and this leads to an Arrhenius conductivity-temperature relationship^31^. The ion hopping increases with an increase of temperature, which enhances the conductivity of the system as activation energy is the minimum energy required for mobile ions in the polymer electrolytes to get excited and starts hopping around inside the polymer matrix. The lower the energy means that it is easier for the mobile ions to move around inside the polymer matrix and it increases the mobility of the mobile ions which leads to increase in the ionic conductivity of the polymer electrolytes^32^. This is in agreement with the ionic conductivity studies showed previously. The decreased *E*_*a*_ and increased ionic conductivity are expected to significantly enhance the reaction kinetics of the DSSCs.

**Figure 3:**
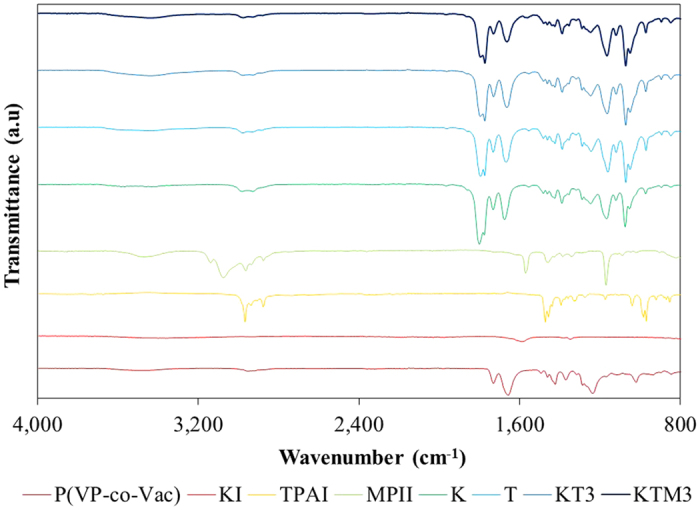
FTIR spectra for pure P(VP-co-VAc), pure KI, pure TPAI, pure MPII and the GPE samples.

The FTIR spectra for pure P(VP-co-VAc), pure KI, pure TPAI, pure MPII and the GPEs are shown in Fig. 3. The main absorption band of P(VP-co-VAc) were summarized and tabulated in Table 2^ ^33,34,35,36,37. As seen in the spectra, the strong band of C = O stretching of PVAc and PVP region of the pure P(VP-co-VAc) which can be seen in 1734 cm^−1^ and 1677 cm^−1^, respectively was found to be shifted to higher wavenumber in the spectrum of all GPE samples. This is the indication of the complexation of the KI and TPAI salt and the MPII ionic liquid in the polymer matrix. The shifting of the bands can also imply that the cations interacted with the strong electron donor group C = O. Similar ion interactions with the carbonyl oxygen of polymers have been reported in literature^38^. Meanwhile, the appearance of the band at 1247 cm^−1^ shown in the figure corresponds to C-N stretching frequency of pure P(VP-co-VAc). It was found to be shifted to higher wavenumbers with the addition of salts and ionic liquid. It is due to the interaction of I^−^ ion from salts and ionic liquid with the strong withdrawing character of the N atom of the C-N in the PVP chain of the P(VP-co-VAc)^39^.

**Figure 4:**
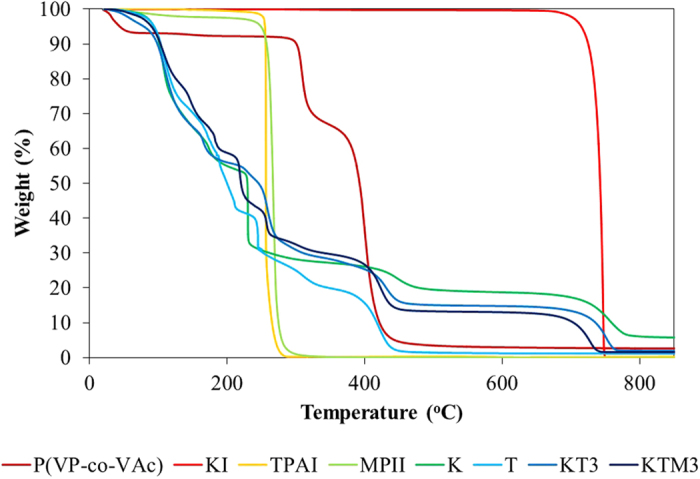
Normalized of dynamic TGA in nitrogen gas for pure P(VP-co-VAc) pure KI, pure TPAI, pure MPII and the GPE samples.

Thermogravimetric analysis of the pure P(VP-co-VAc), pure KI, pure TPAI, pure MPII and the P(VP-co-VAc) based GPEs was investigated at a heating rate of 50.00 °C/min under nitrogen atmosphere and shown in Fig. 4. In the temperature range of 30 °C–100 °C in the GPE sample thermograms, 2–6% of small mass loss can be observed. This small loss was due to the evaporation of low molecular weight substances such as minor impurities and the moisture absorbed by the GPEs. Meanwhile, another mass loss can be observed at temperature range of 100 °C–200 °C which is due to the evaporations of EC, PC, and iodine used in the preparation of the GPEs. In the GPE samples, the EC and PC were found to be evaporating at a temperature which is lower than their initial boiling point. This phenomenon has also been reported in other studies as well^40^. The summarized degradation temperature of the pure P(VP-co-VAc) and the GPE samples are tabulated in Table 3. According to literature studies, pure P(VP-co-VAc) has two stages of degradation which corresponded to the deacetylation of vinyl acetate, PVAc^41^ and the degradation of vinylpyrrolidone, PVP^42^. In these studies, it is represented at T_max1_ = 300 °C and T_max2_ = 385 °C for PVAc and PVP region, respectively and it can be seen in the thermogram of pure P(VP-co-VAc). As observed in Table 3, after the addition of the binary salt and ionic liquid into the system, T_max1_ was found to be decreasing. This shows that the PVAc region in the GPEs samples suffers a decrease in thermal stability upon addition of the KI, TPAI, and MPII. This is most likely due to the complexation that occurred inside the polymer matrix in between the salts and ionic liquids which also shown in FTIR studies. These complexation has probably reduced the crystallinity and softened the backbone of the PVAc region and thus, reducing the decomposition temperature. In contrast, it is observed that PVP region for all the GPEs is increasing in thermal stability. As observed in the table, the addition KI has increases the T_max2_ from 400 °C to 444 °C. This is most likely due to the nature of KI which has very high decomposition temperature as seen in Fig 4. Upon the addition of more TPAI into the system, as seen in sample KT3, the decomposition temperature of the PVP region, T_max2_ decreases from 444 °C to 409 °C. TPAI was observed to have a lower decomposition temperature compared to KI and this could be the reason that has reduced the T_max2_ of the sample KT3. However, the addition of MPII was seen to be able to improve the thermal stability of the GPEs where T_max2_ of KT3 increases from 409 °C–432 °C upon the addition of 15 wt.% of MPII (KTM3). Ionic liquid was known to be able to increase the thermal stability of the polymer electrolytes and it has been reported in literature^43^,^44^ as well. The weight loss at 700 °C–800 °C which can be seen in all of the GPE samples corresponded to the degradation of the KI^45^.

**Figure 5:**
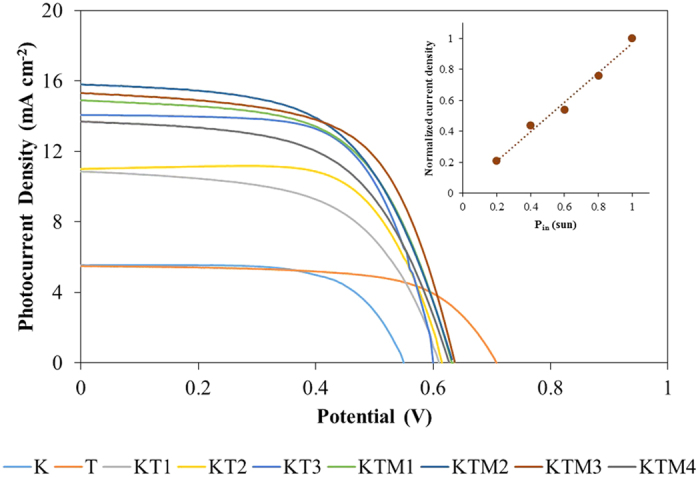
The photocurrent-photovoltage (J-V) characteristics of the different GPE samples. Inset: Normalized J_sc_ values plotted as a function of different light intensities (Pin) of sample KTM3.

**Figure 6:**
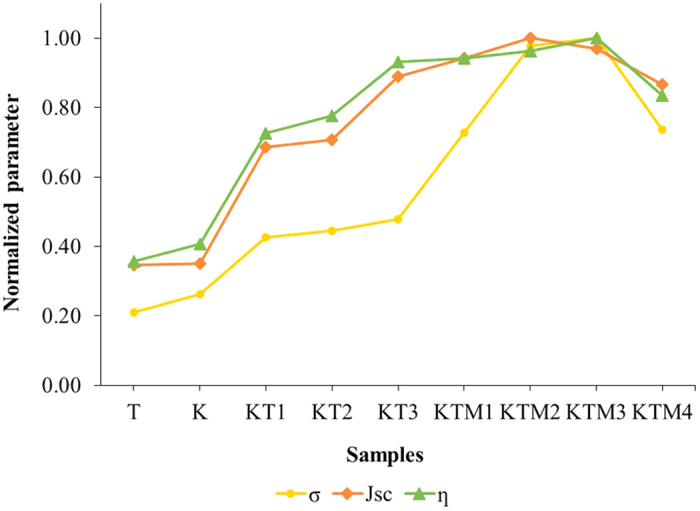
The normalization curves of the ionic conductivity (σ), short-circuit current density (J_sc_) and PCE (*η*) parameters of the DSSCs fabricated with different GPE samples.

The photovoltaic performance of the DSSCs fabricated with the gel polymer electrolytes samples under the simulated solar light of 100 mW cm^−2^ (AM 1.5) was evaluated, and the results are shown in Fig. 5. The calculated photovoltaic parameters of the cells are listed in Table 4. The normalized value of efficiency (η), short-circuit current density (J_sc_) and ionic conductivity of the DSSCs are shown in Fig. 6. Initially, the GPEs with only single salt achieving power conversion efficiencies of 2.11% and 2.42% for TPAI^27^ and KI system, respectively. However, when these two salts were added into the system together, the power conversion efficiency was increased up to 5.53% and there is an abrupt increase of J_sc_ from 5.60 mA cm^−2^ and 5.50 mA cm^−2^ for sample T and sample K, respectively to 14.07 mA cm^−2^ for sample KT3. This is due to the faster transportation rate of the I^−^/I_3_^−^ in the gel polymer electrolyte systems caused by the increase of the ionic conductivity of the GPE samples^46^,^47^. This could be further confirmed by the similar trend of the J_sc_, photovoltaic efficiency and ionic conductivity studies which can be seen in Fig. 6. Typically, electrolytes with smaller cations would have a higher drop in the V_oc_ due to a larger downward shift to the conduction band edge which induced by the adsorption of the smaller cations on the surface of nano-sized TiO_2_ grain^23^,^48^. A similar occurrence is seen in our studies where the V_oc_ of the larger cation, TPA^+^ system was higher (710 V) compared to sample with smaller cation, K^+^ (550 V). In the case of the binary salts systems, as the salts were added together, the V_oc_ would tend to change in the favor of the concentration of the types of salts added. It can be seen in sample KT3 where more KI was incorporated in the GPE, the V_oc_ of the sample was found to be lower compared to the other two sample which having higher percentages of TPAI. The fill factor of the binary salts system was observed to have a value in between the two single salts system. Upon the addition of MPII into the binary salt system, the power conversion efficiency increased to 5.94% in which is mostly due to the increase in V_oc_ and J_sc_ of the system. As mentioned previously, bulkier cations would have a lower downward shift to the conduction band edge which causing it to have higher V_oc_ and with the bulky cation from MPII added into the system it slightly increases the V_oc_. Meanwhile, the slight improvement of the J_sc_ could be due to the increase in the iodide ion conductivity which has been mentioned previously. It might also due to the plasticizing effect that could have decreased the viscosity of the GPE which could lead to an increase in the flow of the mobility of the iodide ion in the polymer matrix^49^. The variation of the normalized current density of the DSSC fabricated with sample KTM3 under different illumination condition was measured and shown in the inset of Fig. 5. The linear shape of the curve shows that there are no mobile ions and electron transport occurring at the Pt and electrode interface that could represent the rate-determining steps in the photo-electrochemical process^50^,^51^.

**Figure 7:**
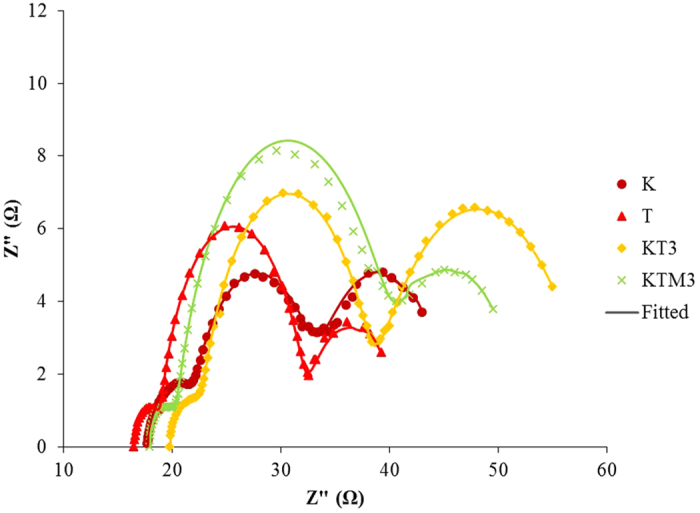
The electrochemical impedance spectra of DSSCs assembled with GPE samples in the forms of Nyquist plot in dark.

The interface resistance-characteristics of a DSSC were usually studied by EIS technique, which is an electrochemical and photo-electrochemical method for analyzing the variations in the impedances associated with different interfaces of a DSSC. Fig. 7 presents the Nyquist plots of the gel polymer electrolytes sample K, sample T, sample KT3, and sample KTM3 in dark with TiO_2_ thickness of 4 μm. In the Nyquist plots of the samples, three distinctive arcs can be observed. At high frequency of 100,000 to 1,000 Hz, the arc observed here corresponded to the charge transfer process at the Pt and electrolyte interface. Meanwhile, the middle frequency arc which is located at 1,000 to 1 Hz corresponds to the electron recombination mechanism in TiO_2_ and the arc at the lower frequency which is located from 1 to 0.1 Hz was attributed to the diffusion in I^−^/I_3_^−^ electrolyte^52^. The intersection of the x-axis and the high-frequency semi-circle is basically interpreted as the sheet resistance of the FTO substrate and it is known as the ohmic serial resistance, R_s_^18^. The R_s_ for sample sample K, sample T, sample KT3, and sample KTM3 is 17.7 Ω, 17.9 Ω, 16.4 Ω and 19.6 Ω, respectively.

**Figure 8:**
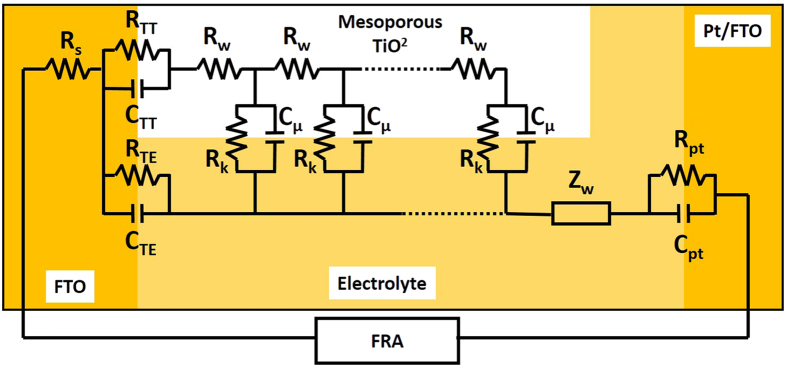
Equivalent circuit model of the DSSCs for electrochemical impedance analysis.

Figure 8 shows the equivalent circuit model used to fit the impedance data^53^. This model was proposed by Bisquert *et al*. in order inspect the transport properties of the injected electrons in the TiO_2_ film and the back electron-reaction with redox species in the gel polymer electrolytes^54^. The data were fitted (represented by lines) with Metrohm Nova software and the parameters used to fit the curves were listed in Table 5. Sample KTM3 also has been run under illuminated sunlight 100 mW cm^−2^ and this sample was labelled as 1 S-KTM3. Some parameters could be estimated from the following equations^54^:


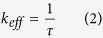



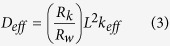



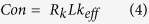



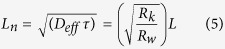


where k_eff_, τ, R_w_, R_k_, L, D_eff_, and L_n_ are the effective rate constant for electron recombination, lifetime of an electron, electron transport resistant in TiO_2_, charge-transfer resistance related to recombination of an electron, thickness of TiO_2_ film, diffusion coefficient and effective diffusion length, respectively.

The value of R_k_ can be estimated approximately from the diameter of the middle arc resistance. Then, from the shape of the central arc, R_k_/R_w_ can be estimated. If the arcs were found to be a true half-circle, then R_k_»R_w_. As observed in the figure, the shape of the central arc of the DSSCs is approximately true half-circle. Thus, R_w_ was able to be obtained from the ratio of R_k_/R_w_ which is normally around 10. Even though the increase in R_k_ implies more recombination, the ratio of R_k_/R_w_ is the important parameter that needs to be focused in this case. This is because higher R_k_/R_w_ basically implies recombination resistance is larger than electron transport resistance. This suggests that there would be lower chances for the recombination of electron with the triiodides of the electrolytes and lower resistance occurred to the electron transport in the TiO_2_ film. This explains the high electron lifetime of the sample in higher R_k_/R_w_^55^. Electron lifetime in the TiO_2_ film can be calculated from Equation 4 where k_eff_ can be obtained from the peak frequency of the middle arc in Fig. 7. The k_eff_ is in an inverse relationship with the electron lifetime in the TiO_2_. The longer τ of the DSSCs indicates more effective suppression of the back reaction between the electrons in its conduction band and the I_3_^−^ ions in the electrolyte. Thus, the increase in τ is the source of the increase of J_sc_ and *η* of the DSSCs fabricated with the P(VP-co-VAc) based GPEs^56^.

On the other hand, in order to clarify the effect of the MPII ionic liquids on the binary salt system on the diffusion coefficient of triiodide in the electrolytes and the photovoltaic performance of the DSSCs, a comparison is made in between the DSSCs of sample KT3 and KTM3. The diffusion coefficient of the sample KTM3 was found to be higher than KT3. This is most probably the reason for the increase in photovoltaic efficiencies of the DSSCs. This increment can be recognized due to the larger number of the diffusion coefficient of triiodide rendering the dye molecule to be able to regenerate more easily resulting in lower recombination rate which leads to increase of J_sc_^57^,^58^,^59^. Normally, the charge transport mechanism in the polymer electrolyte of the DSSCs can be interpreted as Grotthus mechanism which is also known as the electron hopping and ion exchange^60^,^61^,^62^. The increase of the diffusion coefficient could be explainede by the Grotthus mechanism illustrated as below^63^:


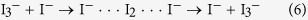


The low conductivity and J_sc_ gel polymer electrolyte could be caused by the average distance between I^−^ ions being too far to transport electrons efficiently. Therefore, when MPII was added into the binary salts gel polymer electrolyte, the imidazolium cations could align the anionic redox couple I^−^/I_3_^−^ inside the polymer matrix by electrostatic interaction, thus the distance between I^−^ and I_3_^−^ would be decreased and higher efficiency electron transport channel could be created. As a result, the ion conduction of the MPII-added GPEs increased and the J_sc_ and *η* was enhanced as well^63^.

**Figure 9:**
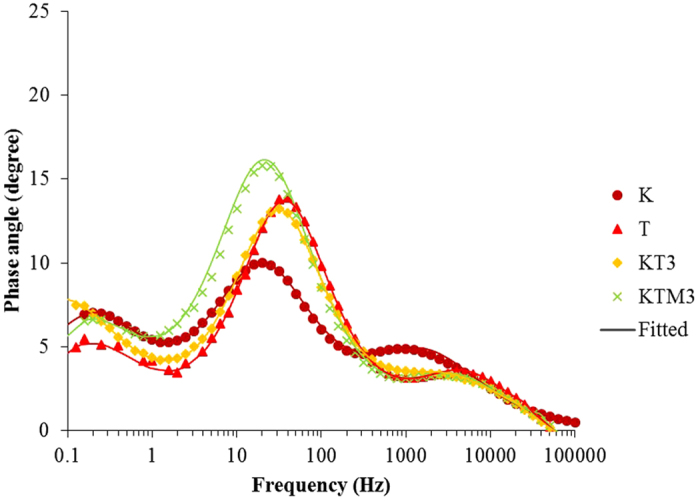
The electrochemical impedance spectra of DSSCs assembled with GPE samples in the forms of Bodes plot in dark.

In the Bode plots in Fig. 9, it can be seen the addition of the MPII ionic liquid shifted the middle frequency peak to lower frequencies. This corresponds to the prolonged electron lifetime. MPII was found to be able to move the conduction band of the TiO2 photoanode positively causing the electrons to be able to recombine with the I_3_^−^ in the electrolyte which causes the electron lifetime at this interface to be prolonged^64^.

**Figure 10:**
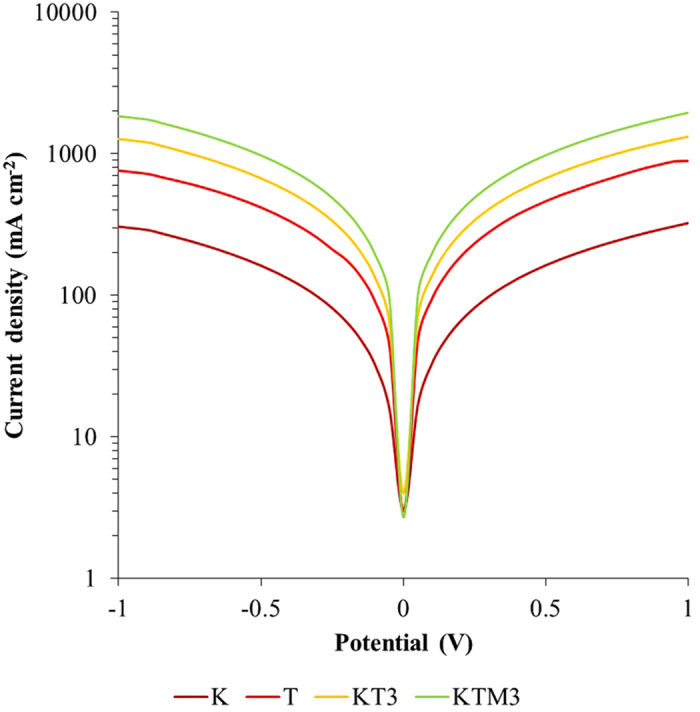
Tafel polarization curves of symmetric Pt cells with different GPE samples.

Tafel-polarization plots were recorded to determine the electrocatalytic activity of the GPE samples with a symmetrical Pt electrode. The results are shown in Fig. 10. In a Tafel plot, a larger slope in the anodic or cathodic branch indicates a higher exchange current density (J_0_). Considering that the pure Tafel region is not observable, therefore, the low field region is used to assess J_0_ variation. The extracted J_0_ has an order of sample T > sample KTM3 > sample KT3 > sample K. J_0_ is inversely proportional to R_ct_ where it can be associated in the following equation^65^:


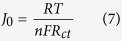


where *R* is the universal gas constant and *T* is the absolute temperature. Apparently, the trend of the *J*_*0*_ matches the order of the results of *R*_*ct*_ in the EIS studies. On the other side, the intersection of the cathodic branch with the Y-axis can be determined as the limiting diffusion current density (*J*_*lim*_), a parameter that depends on the diffusion coefficient (*D*_*n*_) of I^−^/I_3_^−^ redox couple at the counter electrode and electrolyte interface. *J*_*lim*_ is in proportion to *D*_*n*_ where the equation is as following^66^:


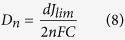


where *d* is the cell gap, *n* is the number of electrons, *F* is the Faraday constant and *C* is the initial concentration of I_3_^−^ ions. Both *J*_*lim*_ and *D*_*n*_ have a sequence of sample KTM3 > sample KT3 > sample T > sample K which indicates that the electrocatalytic activities of the samples increases with the addition of salts at the beginning and then second salts and lastly the addition of the MPII into the system^67^. This explains the trend of the increasing of the PCE of the GPE samples of the J-V studies.

In conclusion, we have prepared and optimized the P(VP-co-VAc) based gel polymer electrolytes by incorporating two salts with two different sizes (KI and TPAI) and MPII ionic liquid. The highest ionic conductivity that was achieved at 4.09 × 10^−3^ S cm^−1^ in sample KTM3 which was developed by KI, TPAI and MPII at an optimum concentration. The activation energy of the same sample was found to be consistent with results of the ionic conductivity as well. The conductivity-temperature (σ-T) relationship for the GPE samples indicates that they are obeying the Arrhenius behavior. The highest power conversion efficiency that was obtained is 5.94% which is from sample KTM3. The EIS test results show that the sample with higher content of bulkier cations would have higher electron lifetime which could lead to an increase in V_oc_ of the sample and the Tafel studies also explained the electrocatalytic studies that affected the PCE of the GPE samples.

## Methods

### Materials

Poly(1-vinylpyrrolidone-co-vinyl acetate) (P(VP-co-VAc)) (Mw, ~50,000 g mol^−1^), ethylene carbonate (EC), propylene carbonate (PC), 1-methyl-3-propylimidazolium iodide (MPII), tetrapropylammonium iodide (TPAI) and sensitizing dye di-tetrabutylamm onium cis-bis(isothiocyanato)bis(2,2′-bipyridyl-4,4′-dicarboxylato)ruthenium(II) (N719) were purchased from Sigma Aldrich. KI and iodine chip (I_2_) were purchase from Friedemann Schmidt Chemical. P(VP-co-VAc), KI, and TPAI were dried in the vacuum oven at 50 °C prior to use.

### Gel polymer electrolytes preparation

The samples were prepared with the composition shown in Table 1. Salts and other additives used for the preparation of the gel electrolytes in this work were dissolved in EC and PC mixture inside small vessels and maintained stirring at 60 °C until complete dissolution. The EC and PC mixture has the weight ratio of 1:1 and the weight of the iodine would be the 10:1 of the molar ratio of the salts to the iodine. After complete dissolution, the copolymer would be added in slowly into the mixture while being stirred at 80 °C for 1 hours. Then, the solution would be cool down to room temperature and the gel network will form and the gel polymer electrolytes were obtained.

### Electrical characteristics of the GPEs

The ionic conductivity of the GPEs was measured with an AC complex impedance spectroscopy with a computer controlled HIOKI 3532-50 LCR Hi-Tester over the frequency range of 50 Hz to 1 MHz at 0.001 V applied voltage. The ionic conductivity were calculated with the following formula:


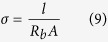


where *σ* is ionic conductivity in S cm^−1^, *l* is the thickness of the thin film sample in cm, *R*_*b*_ is bulk resistance in Ω obtained from Cole–Cole impedance plot and *A* is the surface area of the sample touching on the electrodes in cm^2^. The GPEs were sandwiched between two polished stainless steel electrodes and then used for the conductivity measurements. The thickness of the GPE is controlled with a 0.75-mm-thick Teflon^®^ spacer in between the electrodes. Temperature variation of the conductivity for each sample was obtained by taking measurements at approximately 10 °C intervals in the temperature range 30 °C to 100 °C. At each temperature, the sample was allowed to stabilize for about 30 min before the measurement was taken.

### Preparation of the electrodes

Titanium dioxide (TiO_2_) blocking layers were coated with simple doctor blade methods. FTO-covered glasses were used as the transparent conductive substrates. Prior to the coating process, these substrates were properly cleaned with distilled water and ethanol in an ultrasonic bath. The TiO_2_ paste was prepared by mixing Degussa-P25 (D25) TiO_2_ powders (0.5 g), Triton X-100 (0.035 g) as the dispersing agent and 2 ml of nitric acid (pH = 1). The mixture was then ground for 30 minutes to ensure that the TiO_2_ particles were fully dispersed in the mixture. Then, the mixture was coated on the substrate and sintered at 450 °C for 30 minutes to allow the formation of a mesoporous TiO_2_ film with a mean thickness of 8 μm. The TiO_2_ electrode area was 0.25 cm^2^. Finally, the TiO_2_ photoelectrodes were soaked into a 0.5 mM N719 dye solution in ethanol for 12 h at room temperature and then rinsed in ethanol to remove the unabsorbed dye molecules. Meanwhile, the counter electrode was prepared with a mixture of Chloroplatinic acid solution (H_2_PtCl_6_) and isopropyl alcohol (C_3_H_7_OH) with the weight ratio of 1:1. The mixture was dropped on the conducting surface of the cleaned substrate and air dried. The coated substrates were then sintered with the digital program settings of 100 °C for 5 minutes and followed by 500 °C for 30 minutes. The resulting Pt coated FTO glasses were then cooled to room temperature and gently washed with ethanol. The same process was repeated twice in order to obtain Pt counter electrode with low resistance.

### Fabrication and testing of DSSCs

**Figure 11:**
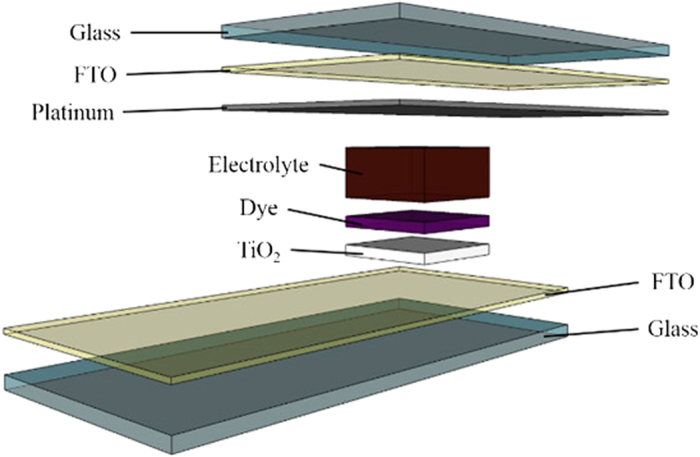
Schematic diagram of the assembled DSSCs.

DSSCs were fabricated in the laboratory as illustrated in Fig. 11. Different GPEs samples were sandwiched in between the TiO_2_ photoelectrode and Pt counter electrode and were sent for photocurrent-voltage (J-V) characteristics under the illumination in the range of 20–100 mW cm^−2^ (P_in_ = 0.2–1.0) simulated sunlight from a Newport LCS-100 Series solar simulator, with a Metrohm Autolab potentiostat (PGSTAT128N). The fill factor (FF) and light-to-electric PCE (*η*) of the cells were calculated according to the following equation:


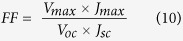



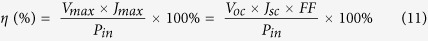


where *J*_*sc*_ is the short-circuit current density (mA cm^−2^), *V*_*oc*_ is the open-circuit voltage (V), *P*_*in*_ is the incident light power, and *J*_*max*_ (mA cm^−2^) and *V*_*max*_ (V) are the current density and voltage in the J-V curves at the point of maximum power output, respectively. The electrochemical impedance spectroscopy was also studied with the same potentiostat in the range of 0.1 Hz – 100 kHz with AC potential of 10 mV. A potential bias equal to open circuit voltage was also applied. The obtained impedance spectra were analyzed and fitted using Metrohm Autolab Nova software with appropriate equivalent circuits. Tafel polarization curves were obtained for different GPEs samples by sandwiching them in between two symmetrical Pt electrodes in the potential range of ±1 V.

## Additional Information

**How to cite this article**: Ming, N. H. *et al*. The potential of incorporation of binary salts and ionic liquid in P(VP-co-VAc) gel polymer electrolyte in electrochemical and photovoltaic performances.. *Sci. Rep*. **6**, 27630; doi: 10.1038/srep27630 (2016).

## Figures and Tables

**Table 1:** The designation and compositions of the GPE samples.

Designations	KI:TPAI:MPII (wt. %)	P(VP-co-VAc) (g)	KI (g)	Pr_4_NI (g)	I_2_(g)	MPII (g)
K	40:0:0	2.694	2.000	0.000	0.306	0.00
T	0:40:0	2.838	0.000	2.000	0.162	0.00
KT1	10:30:0	2.802	0.500	1.500	0.198	0.00
KT2	20:20:0	2.766	1.000	1.000	0.234	0.00
KT3	30:10:0	2.730	1.500	0.500	0.270	0.00
KTM1	30:10:5	2.730	1.500	0.500	0.270	0.25
KTM2	30:10:10	2.730	1.500	0.500	0.270	0.50
KTM3	30:10:15	2.730	1.500	0.500	0.270	0.75
KTM4	30:10:20	2.730	1.500	0.500	0.270	1.00

**Table 2:** FTIR parameters for pure P(VP-co-VAc) copolymer.

Respective bands	Wavenumber (cm^−1^)
O-H stretching	3600 – 3000
C-H stretching	3000 – 2800
C = O carbonyl group (PVAc)	1734
C = O carbonyl group (PVP)	1677
C-N stretching	1446
C-O-C stretching	1300 – 1000
C-H bending (PVAc)	1380
C-H wagging (PVAc)	775
C-H bending (PVP)	1433
C-N stretching (PVP)	1247
C-CH_2_ stretching (PVP)	1171
C-C stretching (PVP)	972
CH_2_ bending (PVP)	846

**Table 3:** The degradation temperature of the pure P(VP-co-VAc) and different GPE samples.

Samples	T_max1_ (°C)	T_max2_ (°C)
Pure P(VP-co-VAc)	310	400
K	230	444
T	245	410
KT3	256	409
KTM3	258	432

**Table 4:** Photovoltaic parameters of the GPE samples under 100 mW cm^−2^ illuminated sunlight.

Electrolytes	V_oc_ (mV)	J_sc_ (mA cm^−2^)	FF (%)	*η* (%)
K	550	5.60	69	2.11
T	710	5.50	62	2.42
KT1	620	10.86	64	4.31
KT2	610	11.18	68	4.61
KT3	600	14.07	65	5.53
KTM1	630	14.90	59	5.60
KTM2	630	15.81	57	5.72
KTM3	640	15.32	61	5.94
KTM4	630	13.70	58	4.96

**Table 5:** The parameters of the equivalent circuits used to fit the EIS impedance data of the DSSCs.

Electrolytes	R_k_ (Ω)	R_w_ (Ω)	R_k_/R_w_ (Ω)	K_eff_ (s^−1^)	τ (ms)	D_eff_ (cm^2^ s^−1^)	Con (Ω cm s^−1^)	R_D_(Ω)	n_s_ (cm^−3^)	L_n_ (μm)
K	13.7	12.6	1.09	16	9.95	2.78 × 10^−6^	0.088	11.5	8.56 × 10^18^	52.62
T	13.9	3.2	4.37	24	6.63	1.68 × 10^−5^	0.133	20.3	5.63 × 10^18^	105.50
KT3	16.5	2.4	6.76	16	9.95	1.73 × 10^−5^	0.106	20	7.11 × 10^18^	131.23
KTM3	20.0	2.1	9.52	15	10.61	2.29 × 10^−5^	0.120	8.6	6.26 × 10^18^	155.73
1 S-KTM3	11.8	1.2	9.83	25	6.37	3.93 × 10^−5^	0.118	6.7	6.36 × 10^18^	158.24

## References

[b1] Bella F., Ozzello E. D., Sacco A., Bianco S. & Bongiovanni R. Polymer electrolytes for dye-sensitized solar cells prepared by photopolymerization of PEG-based oligomers. Int. J. Hydrogen Energy 39, 3036–3045 (2014).

[b2] Bella F., Sacco A., Pugliese D., Laurenti M. & Bianco S. Additives and salts for dye-sensitized solar cells electrolytes: what is the best choice? J. Power Sources 264, 333–343 (2014).

[b3] O’Regan B. & Grätzel M. A low-cost, high-efficiency solar cell based on dye-sensitized colloidal TiO2 films. Nature 353, 737–740 (1991).

[b4] Boonsin R. . Dye-sensitized solar cell with poly(acrylic acid-co-acrylonitrile)-based gel polymer electrolyte. Mater. Chem. Phys. 132, 993–998 (2012).

[b5] Lan J.-L., Wei T.-C., Feng S.-P., Wan C.-C. & Cao G. Effects of Iodine Content in the Electrolyte on the Charge Transfer and Power Conversion Efficiency of Dye-Sensitized Solar Cells under Low Light Intensities. J. Phys. Chem. C 116, 25727–25733 (2012).

[b6] Hamadanian M., Safaei-Ghomi J., Hosseinpour M., Masoomi R. & Jabbari V. Uses of new natural dye photosensitizers in fabrication of high potential dye-sensitized solar cells (DSSCs). Mater. Sci. Semicond. Process. 27, 733–739 (2014).

[b7] Kumar C. H. P. . New ruthenium complexes (Ru[3 + 2 + 1]) bearing π-extended 4-methylstyryl terpyridine and unsymmetrical bipyridine ligands for {DSSC} applications. Inorganica Chim. Acta 435, 46–52 (2015).

[b8] Park N., Chang S., Lagemaat J., Van De, Kim K. & Frank A. J. Effect of Cations on the Open-Circuit Photovoltage and the Charge-Injection Efficiency of Dye-Sensitized Nanocrystalline Rutile TiO 2 Films. 21, 985–988 (2000).

[b9] Xu P., Tang Q., He B., Li Q. & Chen H. Transmission booster from SiO2 incorporated TiO2 crystallites: Enhanced conversion efficiency in dye-sensitized solar cells. Electrochim. Acta 134, 281–286 (2014).

[b10] Wang M. . Counter electrodes from polyaniline−graphene complex/graphene oxide multilayers for dye−sensitized solar cells. Electrochim. Acta 137, 175–182 (2014).

[b11] He B., Tang Q., Meng X. & Yu L. Poly(vinylidene fluoride)–implanted cobalt–platinum alloy counter electrodes for dye–sensitized solar cells. Electrochim. Acta 147, 209–215 (2014).

[b12] Seo S.-J., Cha H.-J., Kang Y. S. & Kang M.-S. Printable ternary component polymer-gel electrolytes for long-term stable dye-sensitized solar cells. Electrochim. Acta 145, 217–223 (2014).

[b13] Ahmad S., Guillen E., Kavan L., Gratzel M. & Nazeeruddin M. K. Metal free sensitizer and catalyst for dye sensitized solar cells. Energy Environ. Sci. 6, 3439–3466 (2013).

[b14] Vittadello M. . Iodide-conducting polymer electrolytes based on poly-ethylene glycol and MgI2: Synthesis and structural characterization. Electrochim. Acta 57, 112–122 (2011).

[b15] Ahmad S., Deepa M. & Agnihotry S. A. Effect of salts on the fumed silica-based composite polymer electrolytes. Sol. Energy Mater. Sol. Cells 92, 184–189 (2008).

[b16] Lim S. J., Kang Y. S. & Kim D.-W. Dye-sensitized solar cells with quasi-solid-state cross-linked polymer electrolytes containing aluminum oxide. Electrochim. Acta 56, 2031–2035 (2011).

[b17] Nair J. R. . Truly quasi-solid-state lithium cells utilizing carbonate free polymer electrolytes on engineered LiFePO4. Electrochim. Acta 199, 172–179 (2016).

[b18] Wang C., Wang L., Shi Y., Zhang H. & Ma T. Printable electrolytes for highly efficient quasi-solid-state dye-sensitized solar cells. Electrochim. Acta 91, 302–306 (2013).

[b19] Mayumi S. . Highly stable dye-sensitized solar cells with quasi-solid-state electrolyte based on Flemion. Sol. Energy 110, 648–655 (2014).

[b20] Bandara T. M. W. J., Svensson T., Dissanayake M. A. K. L. & Furlani M. Tetrahexylammonium Iodide Containing Solid and Gel Polymer Electrolytes for Dye Sensitized Solar Cells. Energy Procedia 14, 1 (2012).

[b21] Saikia D., Chen-Yang Y. W., Chen Y. T., Li Y. K. & Lin S. I. Investigation of ionic conductivity of composite gel polymer electrolyte membranes based on P(VDF-HFP), LiClO4 and silica aerogel for lithium ion battery. Desalination 234, 24–32 (2008).

[b22] Kumar A., Logapperumal S., Sharma R., Das M. K. & Kar K. K. Li-ion transport, structural and thermal studies on lithium triflate and barium titanate incorporated poly(vinylidene fluoride-co-hexafluoropropene) based polymer electrolyte. Solid State Ionics 289, 150–158 (2016).

[b23] Dissanayake M. a. K. L. .. Efficiency enhancement by mixed cation effect in dye-sensitized solar cells with PAN based gel polymer electrolyte. J. Photochem. Photobiol. A Chem. 246, 29–35 (2012).

[b24] Ahmad S. & Deepa M. Ionogels encompassing ionic liquid with liquid like performance preferable for fast solid state electrochromic devices. Electrochem. commun. 9, 1635–1638 (2007).

[b25] Gerbaldi C. . UV-cured polymer electrolytes encompassing hydrophobic room temperature ionic liquid for lithium batteries. J. Power Sources 195, 1706–1713 (2010).

[b26] Li P. J. . The application of P(MMA-co-MAA)/PEG polyblend gel electrolyte in quasi-solid state dye-sensitized solar cell at higher temperature. Electrochim. Acta 53, 903–908 (2007).

[b27] Ng H. M., Ramesh S. & Ramesh K. Exploration on the P(VP-co-VAc) copolymer based gel polymer electrolytes doped with quaternary ammonium iodide salt for DSSC applications: Electrochemical behaviors and photovoltaic performances. Org. Electron. physics, Mater. Appl. (2015). doi: 10.1016/j.orgel.2015.03.020

[b28] Agarwala S. . Co-existence of LiI and KI in filler-free, quasi-solid-state electrolyte for efficient and stable dye-sensitized solar cell. J. Power Sources 196, 1651–1656 (2011).

[b29] Sekhon S. S., Krishnan P., Singh B., Yamada K. & Kim C. S. Proton conducting membrane containing room temperature ionic liquid. Electrochim. Acta 52, 1639–1644 (2006).

[b30] Tang Z. . Preparation of PAA-g-CTAB/PANI polymer based gel-electrolyte and the application in quasi-solid-state dye-sensitized solar cells. Electrochim. Acta 58, 52–57 (2011).

[b31] Nath a. K. & Kumar a. Scaling of AC conductivity, electrochemical and thermal properties of ionic liquid based polymer nanocomposite electrolytes. Electrochim. Acta 129, 177–186 (2014).

[b32] Yang C.-C., Wey J.-Y., Liou T.-H., Li Y. J. & Shih J.-Y. A quasi solid state dye-sensitized solar cell based on poly(vinylidenefluoride-co-hexafluoropropylene)/SBA-15 nanocomposite membrane. Mater. Chem. Phys. 132, 431–437 (2012).

[b33] Lee C.-P., Lin L.-Y., Vittal R. & Ho K.-C. Favorable effects of titanium nitride or its thermally treated version in a gel electrolyte for a quasi-solid-state dye-sensitized solar cell. J. Power Sources 196, 1665–1670 (2011).

[b34] Ulaganathan M., Nithya R., Rajendran S. & Raghu S. Li-ion conduction on nanofiller incorporated PVdF-co-HFP based composite polymer blend electrolytes for flexible battery applications. Solid State Ionics 218, 7–12 (2012).

[b35] Ramya C. S., Selvasekarapandian S., Hirankumar G., Savitha T. & Angelo P. C. Investigation on dielectric relaxations of PVP–NH4SCN polymer electrolyte. J. Non. Cryst. Solids 354, 1494–1502 (2008).

[b36] Kumar D. A., Selvasekarapandian S., Baskaran R., Savitha T. & Nithya H. Thermal, vibrational and Ac impedance studies on proton conducting polymer electrolytes based on poly(vinyl acetate). J. Non. Cryst. Solids 358, 531–536 (2012).

[b37] Ravi M., Kiran Kumar K., Madhu Mohan V. & Narasimha Rao V. V. R. Effect of nano TiO2 filler on the structural and electrical properties of PVP based polymer electrolyte films. Polym. Test. 33, 152–160 (2014).

[b38] Wieczorek W. & Stevens J. R. Impedance Spectroscopy and Phase Structure of Polyether - Poly (methyl methacrylate)–LiCF 3 SO 3 Blend-Based Electrolytes. J. Phys. Chem. B 101, 1529–1534 (1997).

[b39] Ramya C. S., Selvasekarapandian S., Savitha T., Hirankumar G. & Angelo P. C. Vibrational and impedance spectroscopic study on PVP–NH4SCN based polymer electrolytes. Phys. B Condens. Matter 393, 11–17 (2007).

[b40] Kim K.-S., Park S.-Y., Yeon S.-H. & Lee H. N-Butyl-N-methylmorpholinium bis(trifluoromethanesulfonyl)imide–PVdF(HFP) gel electrolytes. Electrochim. Acta 50, 5673–5678 (2005).

[b41] Bianco G. . Thermal stability of poly(N-vinyl-2-pyrrolidone-co-methacrylic acid) copolymers in inert atmosphere. Polym. Degrad. Stab. 80, 567–574 (2003).

[b42] McNeill I. C., Ahmed S. & Memetea L. Thermal degradation of vinyl acetate-methacrylic acid copolymer and the homopolymers. II. Thermal analysis studies. Polym. Degrad. Stab. 48, 89–97 (1995).

[b43] Lin B. . Ionic liquid-tethered Graphene Oxide/Ionic Liquid Electrolytes for Highly Efficient Dye Sensitized Solar Cells. Electrochim. Acta (2014). doi: 10.1016/j.electacta.2014.03.064

[b44] Costa L. T. . Polymer electrolytes based on poly(ethylene glycol) dimethyl ether and the ionic liquid 1-butyl-3-methylimidazolium hexafluorophosphate: Preparation, physico-chemical characterization, and theoretical study. Electrochim. Acta 53, 1568–1574 (2007).

[b45] Zaccaron C. M., Oliveira R. V. B., Guiotoku M., Pires A. T. N. & Soldi V. Blends of hydroxypropyl methylcellulose and poly(1-vinylpyrrolidone-co-vinyl acetate): Miscibility and thermal stability. Polym. Degrad. Stab. 90, 21–27 (2005).

[b46] Bandara T. M. W. J. . Quasi solid state polymer electrolyte with binary iodide salts for photo-electrochemical solar cells. Int. J. Hydrogen Energy 39, 2997–3004 (2014).

[b47] Dissanayake M. a. K. L., Rupasinghe W. N. S., Seneviratne V. a. & Thotawatthage C. a. & Senadeera, G. K. R. Optimization of iodide ion conductivity and nano filler effect for efficiency enhancement in polyethylene oxide (PEO) based dye sensitized solar cells. Electrochim. Acta 145, 319–326 (2014).

[b48] Shi Y., Wang Y., Zhang M. & Dong X. Influences of cation charge density on the photovoltaic performance of dye-sensitized solar cells: lithium, sodium, potassium, and dimethylimidazolium. Phys. Chem. Chem. Phys. 13, 14590–7 (2011). 21769357 10.1039/c1cp21020c

[b49] Liew C.-W., Ramesh S. & Arof a. K. Good prospect of ionic liquid based-poly(vinyl alcohol) polymer electrolytes for supercapacitors with excellent electrical, electrochemical and thermal properties. Int. J. Hydrogen Energy 39, 2953–2963 (2014).

[b50] Chiappone A. . Structure-Performance Correlation of Nanocellulose-Based Polymer Electrolytes for Efficient Quasi-solid DSSCs. ChemElectroChem 1, 1350–1358 (2014).

[b51] Salvador G. P. . New insights in long-term photovoltaic performance characterization of cellulose-based gel electrolytes for stable dye-sensitized solar cells. Electrochim. Acta 146, 44–51 (2014).

[b52] Xiao S., Cui J., Yi P., Yang Y. & Guo X. Insight into electrochemical properties of Co3O4–modified magnetic polymer electrolyte. Electrochim. Acta 144, 221–227 (2014).

[b53] Lin C.-Y. . Highly efficient dye-sensitized solar cell with a ZnO nanosheet-based photoanode. Energy Environ. Sci. 4, 3448 (2011).

[b54] Adachi M., Sakamoto M., Jiu J., Ogata Y. & Isoda S. Determination of parameters of electron transport in dye-sensitized solar cells using electrochemical impedance spectroscopy. J. Phys. Chem. B 110, 13872–80 (2006). 16836336 10.1021/jp061693u

[b55] Chen H.-W. . Electrophoretic deposition of TiO2 film on titanium foil for a flexible dye-sensitized solar cell. Electrochim. Acta 56, 7991–7998 (2010).

[b56] Kumar E. N. . High performance dye-sensitized solar cells with record open circuit voltage using tin oxide nanoflowers developed by electrospinning. Energy Environ. Sci. 5, 5401 (2012).

[b57] Li C. . New Class of Ionic Liquids for Dye-Sensitized Solar Cells. (2015).

[b58] Senadeera G. & De Silva N. Efficient quasi-solid dye sensitized solar cells employing molten salt electrolyte. Sri Lankan J. Phys. 7, 15–22 (2008).

[b59] Fredin K. *Studies of Charge Transport Processes in Dye-Sensitized Solar Cells*. at <http://www.diva-portal.org/smash/record.jsf?pid=diva2:12288> (2007)

[b60] Hu M. . Enhancement of monobasal solid-state dye-sensitized solar cells with polymer electrolyte assembling imidazolium iodide-functionalized silica nanoparticles. J. Power Sources 248, 283–288 (2014).

[b61] Wu J. . Influence of solvent on the poly (acrylic acid)-oligo-(ethylene glycol) polymer gel electrolyte and the performance of quasi-solid-state dye-sensitized solar cells. Electrochim. Acta 52, 7128–7135 (2007).

[b62] Lan Z. . Influence of ionic additives NaI/I2 on the properties of polymer gel electrolyte and performance of quasi-solid-state dye-sensitized solar cells. Electrochim. Acta 53, 2296–2301 (2008).

[b63] Boschloo G., Hagfeldt A. & Spectus C. O. N. Characteristics of the Iodide/Triiodide Redox Mediator in Dye-Sensitized Solar Cells. Acc. Chem. Res. 42, 1819–1826 (2009). 19845388 10.1021/ar900138m

[b64] Cui Y. . Improved performance using a plasticized polymer electrolyte for quasi-solid state dye-sensitized solar cells. Electrochim. Acta 74, 194–200 (2012).

[b65] Wu C. . Electrochemical characterization of a novel iodine-free electrolyte for dye-sensitized solar cell. Electrochim. Acta 71, 33–38 (2012).

[b66] Wang M. . CoS supersedes Pt as efficient electrocatalyst for triiodide reduction in dye-sensitized solar cells. J. Am. Chem. Soc. 131, 15976–7 (2009). 19845335 10.1021/ja905970y

[b67] Park, S. H. . Poly(3,4-Ethylenedioxythiophene) Inverse Opal Electrode Fabricated from Poly(3,4-Ethylenedioxythiophene):Poly(Styrene Sulfonate)-Filled Polystyrene Template for Dye-Sensitized Solar Cells. Electrochim. Acta 137, 661–667 (2014).

